# Spatiotemporal epidemiology of cryptosporidiosis in the Republic of Ireland, 2008–2017: development of a space–time “cluster recurrence” index

**DOI:** 10.1186/s12879-021-06598-3

**Published:** 2021-08-28

**Authors:** M. Boudou, E. Cleary, C. ÓhAiseadha, P. Garvey, P. McKeown, J. O’Dwyer, Paul Hynds

**Affiliations:** 1 Environmental Sustainability and Health Institute (ESHI), Technological University Dublin, Greenway Hub, Grangegorman, Dublin 7, D07 H6K8 Republic of Ireland; 2 Department of Public Health, Health Service Executive (HSE), Dr. Steevens’ Hospital, Dublin 8, Republic of Ireland; 3 Health Protection Surveillance Centre, 25 Middle Gardiner Street, Dublin 1, Republic of Ireland; 4 School of Biological, Earth and Environmental Sciences, Environmental Research Institute (ERI), University College Cork, Cork, Republic of Ireland; 5 Irish Centre for Research in Applied Geosciences (iCRAG), University College Dublin, Dublin 4, Republic of Ireland

**Keywords:** Cryptosporidiosis, *Cryptosporidium*, Spatiotemporal epidemiology, Clustering, Space–time scanning, Seasonality

## Abstract

**Background:**

Ireland frequently reports the highest annual Crude Incidence Rates (CIRs) of cryptosporidiosis in the EU, with national CIRs up to ten times the EU average. Accordingly, the current study sought to examine the spatiotemporal trends associated with this potentially severe protozoan infection.

**Methods:**

Overall, 4509 cases of infection from January 2008 to December 2017 were geo-referenced to a Census Small Area (SA), with an ensemble of geo-statistical approaches including seasonal decomposition, Local Moran’s *I*, and space–time scanning used to elucidate spatiotemporal patterns of infection.

**Results:**

One or more confirmed cases were notified in 3413 of 18,641 Census SAs (18.3%), with highest case numbers occurring in the 0–5-year range (n = 2672, 59.3%). Sporadic cases were more likely male (OR 1.4) and rural (OR 2.4), with outbreak-related cases more likely female (OR 1.4) and urban (OR 1.5). Altogether, 55 space–time clusters (≥ 10 confirmed cases) of sporadic infection were detected, with three “high recurrence” regions identified; no large urban conurbations were present within recurrent clusters.

**Conclusions:**

Spatiotemporal analysis represents an important indicator of infection patterns, enabling targeted epidemiological intervention and surveillance. Presented results may also be used to further understand the sources, pathways, receptors, and thus mechanisms of cryptosporidiosis in Ireland.

**Supplementary Information:**

The online version contains supplementary material available at 10.1186/s12879-021-06598-3.

## Introduction

*Cryptosporidium* is an oocyst-forming protozoan parasite first identified as a causative agent of gastrointestinal infection in the mid-1970s [1]. Cryptosporidiosis is associated with a wide range of symptoms including watery diarrhoea, weight loss, vomiting, abdominal pain, nausea and fever [2]. In the most severe cases, infection may lead to acute dehydration and death, particularly among immuno-compromised individuals, including children aged ≤ 5 years, the elderly (≥ 65) and patients with underlying health conditions (i.e., immunosuppressed) [3]. To date, approximately 40 genetically distinct *Cryptosporidium* species have been identified, with *C. parvum* and *C. hominis* the most frequently confirmed species among cases of human infection [4]. Transmission typically occurs via the faecal-oral route through consumption of contaminated water or food, in addition to direct human-animal contact and exposure to contaminated environments including recreational water [2, 5–7]. A previous experimental study of healthy adult volunteers indicated that ingestion of 30 oocysts is sufficient to initiate infection, with a significantly lower threshold dose (≈ 10 oocysts) associated with specific *C. hominis* and *C. parvum* strains [5].

Cryptosporidiosis occurs in both rural and urban environments, with several studies indicating that *C. hominis* is more frequent in urban areas (due to increased rates of person-to-person transmission) while *C. parvum* predominates in rural areas [7]. Environmental transmission in rural areas represents a particular concern due to the ability of oocysts to survive for prolonged periods in the natural environment (e.g., soil, water) due to temperature buffering and high humidity [8].

Human cryptosporidiosis became a notifiable disease in Ireland on January 1st 2004 under the Infectious Diseases (Amendment) (No. 3) Regulations 2003 (S.I. 707 of 2003). As such, all medical practitioners are required to notify the regional Medical Officer of Health (MOH)/Director of Public Health of all confirmed cases. According to the most recent European Centre for Disease Prevention and Control (ECDC) report, Ireland consistently reports the highest Crude Incidence Rates (CIR) of confirmed cryptosporidiosis infection in the European Union [9]. For example, during 2017 Ireland reported a cryptosporidiosis CIR of 12.0/100,000 residents, compared with an EU mean CIR of 3.2/100,000 (including 15 member states with national notification rates < 1/100,000) [10]. Nationally, cryptosporidiosis represents the most frequently reported protozoan infection, with CIRs having remained relatively consistent over the past decade, ranging from 11.0/100,000 in 2004 to 13.2/100,000 in 2018 [10]. Unlike other gastroenteric infections (e.g., giardiasis), cryptosporidiosis in Ireland is primarily associated with domestic (indigenous) exposure and transmission. For example, 81% (556/629) of confirmed cases during 2018 were identified as sporadic domestic cases, 12% (n = 73) were associated with a recognised cluster/outbreak, while travel-related cases accounted for 7% (n = 43) of the total case number [10]. The largest Irish cryptosporidiosis outbreak to date was attributed to *C. hominis* and occurred in the west of Ireland during March/April 2007. This was concentrated around Galway city, with at least 242 confirmed cases caused by municipal wastewater ingress to Lough Corrib, a lake employed for public water supply in the region [11]. The economic and human health burden accruing from events like the “Galway outbreak”, recently estimated at approximately €19 million [11] coupled with the high baseline incidence of cryptosporidiosis, create a need for a greater understanding of the sources and transmission routes for the disease.

While several studies have examined the likely routes of exposure to *Cryptosporidium* spp. in Ireland [e.g., 12,13], few epidemiological investigations of the spatiotemporal dynamics of confirmed cryptosporidiosis infection has been undertaken. This represents a significant knowledge gap with respect to understanding pathogen sources and pathways, particularly in light of the endemic nature of cryptosporidiosis in Ireland. An improved mechanistic understanding of infection occurrence would enable earlier detection, enhanced surveillance, and more focused public-health and healthcare policies. The current study sought to explore the temporal and spatial patterns of domestically acquired (sporadic and outbreak-related) cases of cryptosporidiosis in Ireland via identification of infection clustering. To accurately describe the epidemiological patterns of this important zoonotic parasite, the study integrated several modelling approaches including seasonal decomposition, spatial autocorrelation (Anselin Local Moran’s *I*), hot-spot analysis (Getis-Ord Gi*) and space–time scanning with a large georeferenced dataset of confirmed cryptosporidiosis cases (n = 4509) over a 10-year period (2008–2017). To the authors’ knowledge, this represents the first spatio-temporal study of its kind in Ireland, which as previously described, exhibits the highest national cryptosporidiosis infection CIRs in the EU.

## Methods

### Data collection and processing

Irreversibly anonymised cases of cryptosporidiosis reported by regional departments of public health between 1st January 2008 and 31st December 2017 were provided from the national Computerised Infectious Disease Reporting (CIDR) database. Data prior to 2008 were excluded to avoid potential bias being introduced by the large number of cases reported during the 2007 Galway outbreak. All confirmed cases, including patient-specific data fields [age, gender, date of reporting, and case outcome (severity)] were geo-spatially linked to the geographical centroid of their associated Census Small Area (SA) (the smallest administrative unit currently employed for census reporting in Ireland) using the Health Service Executive (HSE) Health Intelligence Unit’s geocoding tools. Sporadic, outbreak-related, and travel-related (non-outbreak) cases were defined and discretized for analyses. Outbreak-related cases are defined as confirmed cases with an attached “CIDR outbreak ID”, used for identifying cases associated with a recognised infection outbreak or cluster. Travel-related cases are specifically categorised for purposes of analytical exclusion or adjustment (i.e., national reporting) and defined as any patient self-reporting travel outside of Ireland within the likely incubation period. Sporadic cases were subsequently delineated via exclusion of the two previous categories from the total case dataset. All case data and analyses were granted full research ethics approval by the Royal College of Physicians of Ireland Research Ethics Committee (RCPI RECSAF_84).

**Fig. 1:**
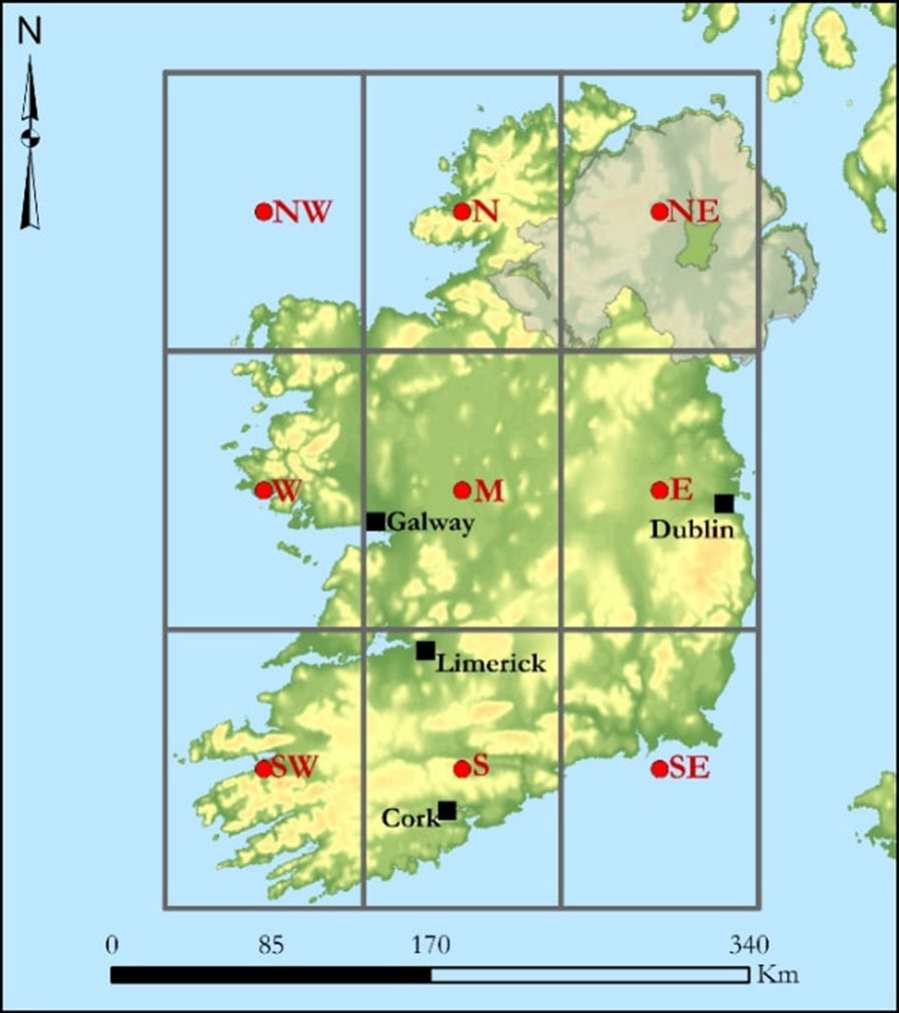
Geographical zonation of the Republic of Ireland

As cryptosporidiosis in Ireland is most prevalent among children ≤ 5 years of age and in rural areas [10], specific analyses were undertaken with respect to case age (≤ 5 years, ≥ 6 years) and land-use classification (rural/urban). The Central Statistics Office (CSO) Census of 2011 and 2016 were used to extract Electoral Division (ED)- and SA-specific human population counts, permitting calculation of cryptosporidiosis incidence rates at both spatial (administrative unit) scales. The CSO’s 14 urban/rural categories were used to classify each spatial unit as rural or urban. Population density and settlement size were employed to verify all classifications. For reporting purposes within the current article, Ireland has been delineated into eight distinct geographical zones (Fig. 1). Zone NE (corresponding to Northern Ireland) is located outside Irish public health legislative jurisdiction and was not included for analyses. Pearsons χ^2^ test with Yates’ continuity corrections and Fisher’s exact test (where any cell had < 5 cases) were used to test for association between categorical case classifications.

### Seasonal decomposition

Seasonal decomposition was carried out using Seasonal and Trend (STL) decomposition via the LOESS (Locally Estimated Scatterplot Smoothing) method on different subsets of the case dataset, e.g., sporadic cases, outbreak-related cases, cases in children ≤ 5 years of age, cases in people ≥ 6 years of age, travel-related cases and cases in urban vs. rural areas. The monthly incidence of infection was calculated for each case sub-category. The STL method decomposes incidence data (*Y*_*v*_) time-series into three separate component series: seasonal variation (*S*_*v*_), overall trend over time (*T*_*v*_) and residuals (*R*_*v*_), whereby the incidence data is equal to the sum of all three trends denoted by [14]:

$$Y_{v} = \, T_{v} + \, S_{v} + \, R_{v}$$

An additive seasonal decomposition formula was used, as opposed to multiplicative, to remove seasonality (*S*_*v*_) and trend (T_v_) from the overall time-series (*Y*_*v*_) and filter random variation from long-term trends given by the residuals (*R*_*v*_) so that: Residuals (*R*_*v*_) = Time series (*Y*_*v*_) − Seasonal trend (*S*_*v*_) − Trend (*T*_*v*_).

### Spatial autocorrelation

The total number of sporadic cryptosporidiosis cases, sporadic cases in children ≤ 5, people aged ≥ 6 years, and outbreak-related cases were mapped to individual SA centroids. Age-adjusted infection rates within each sub-category were calculated at both SA and ED level, based on 2011/2016 census data. Outbreak-related infection rates were calculated as a proportion of overall cases within each SA and ED. Data aggregation and infection rate calculation were carried out in R statistical software version 3.6.0 (R Foundation for Statistical Computing, Vienna, Austria). Anselin Local Moran’s *I* was employed for spatial autocorrelation.

Anselin Local Moran’s *I* focuses on the relationship of individual features with nearby features and assigns clusters based on variance assigned to individual spatial units, thus negating the assumption underlying the Global Moran’s *I* statistic that a single statistic appropriately accounts for clustering and dispersion of the spatial predominance of infection across the entire study area [15]. The Anselin Local Moran’s *I* statistic is calculated by generating a neighbour list of spatially proximal SAs or EDs and calculating spatial autocorrelation of similar infection rates as a function of distance bands, thus identifying localised clusters which are correlated based on the variance assigned to all individual spatial units [15, 16]. Clusters of high-high (H–H) and low–low (L–L) infection, and outliers of high–low (H–L) and low high (L–H) infection are subsequently identified. Local Moran’s *I* statistics were calculated using the cluster and analysis tool in ArcGIS version 10.6 (ESRI, Redlands, California) which generates a Moran’s I statistic, z-score and pseudo p-value for each spatial unit. A positive I value is indicative of spatial units with a high or low infection rate, surrounded by SAs or EDs with similarly high or low infection rates. Conversely, a negative I value indicates outliers of infection where an SA or ED with a high rate of infection is surrounded by SAs or EDs with low rates of infection, and vice versa [15].

### Hot-spot analysis (Getis-Ord GI*)

Hot-spot analysis was carried out for all sporadic cases, sporadic cases among children ≤ 5, cases ≥ 6 years, and outbreak cases by calculating spatially specific Getis-Ord GI* statistics in ArcGIS. The Getis-Ord Gi* statistic is calculated for each feature (SA or ED) in the dataset, generating a unit-specific z-score and *p*-value, used to statistically determine significant spatial clustering of features in the dataset [17]. Statistically significant clusters are clusters which have high values surrounded by SAs or EDs with similarly high values, and vice versa [18]. Hot- and cold-spots of infection are determined based on the spatial proximity of high/low values statistically similar to neighbouring features. Compared with Anselin local Moran’s *I* statistics, clusters based on the Getis-Ord GI* statistic are determined by comparing the sum of local features and their neighbours with the overall sum of all features. Getis-Ord GI* statistics were used to examine whether differing statistical analyses of spatial clustering of infection between spatial units yield varying results. The Getis-Ord GI* statistic is given as [18]:

$$G_{i}^{*} = \frac{{\sum\limits_{j = 1}^{n} {w_{i,j} x_{j} - \overline{X}\sum\limits_{j = 1}^{n} {w_{i,j} } } }}{{S\sqrt {\frac{{\left[ {n\sum\limits_{j = 1}^{n} {w_{i,j}^{2} } - \left( {\sum\limits_{j = 1}^{n} {w_{i,j} } } \right)^{2} } \right]}}{n - 1}} }}$$

where χ_j_ is the attribute value for feature *j*, *w*_*i,j*_ is the spatial weight between feature *i* and *j*, *n* is equal to the total number of features and:

$$\begin{gathered} \overline{X} = \frac{{\sum\limits_{j = 1}^{n} {x_{j} } }}{n} \hfill \\ S = \sqrt {\frac{{\sum\limits_{j = 1}^{n} {x_{j}^{2} } }}{n} - \left( {\overline{X}} \right)^{2} } \hfill \\ \end{gathered}$$

### Space–time scanning

Space–time scanning was undertaken using SaTScan v9.6 software (Kulldorf and Information Management Services, Inc., MA, USA). SaTScan detects spatial clusters of areal units (i.e., SAs/EDs) by imposing an infinite number of overlapping circular (or elliptical) scanning windows of predetermined sizes across a defined geographic area [19]. Temporal clusters were simultaneously assessed using the scan statistic, which includes an infinite number of overlapping cylindrical windows defined by a base (spatial scan statistic) and height (temporal scan statistic) [20].

A discrete Poisson model was employed for space–time scanning to account for the high-resolution spatial scale (n = 18,488 SAs), resulting in high zero/one inflation (i.e., high numbers of SAs with 0 or 1 case). A case threshold of 10 cases (minimum) per cluster was selected to ensure that identified clusters were significant i.e., avoidance of single household clusters. Similarly, a maximum of 10% of population at risk (PAR) was employed concurrently with a maximum cluster radius of 50 kms to account for low case numbers within individual Small Areas. Data were aggregated at a monthly scale, with maximum cluster duration set to 3 months to account for known seasonal variation of cryptosporidiosis in Ireland.

SaTScan analyses produce two primary outputs; a spatial cluster location(s) (cluster centroid and diameter) and descriptive cluster data (start/end dates, total population, number of observed-expected cases, relative risk, and p-value). The authors have developed a novel mapping approach for representing SaTScan results, whereby all significant clusters (p < 0.05) are selected and mapped in ArcGIS (ArcGIS 10.6), with binary cluster location [i.e., Cluster Membership (0/1)] for annual space–time scans summed at the CSO SA scale. The final mapping provides a “cluster recurrence” index ranging from 0 to 10 (i.e., annual absence/presence of cluster over 10-year study period).

## Results

### Occurrence of cryptosporidiosis infection in the Republic of Ireland (2008–2017)

**Fig. 2:**
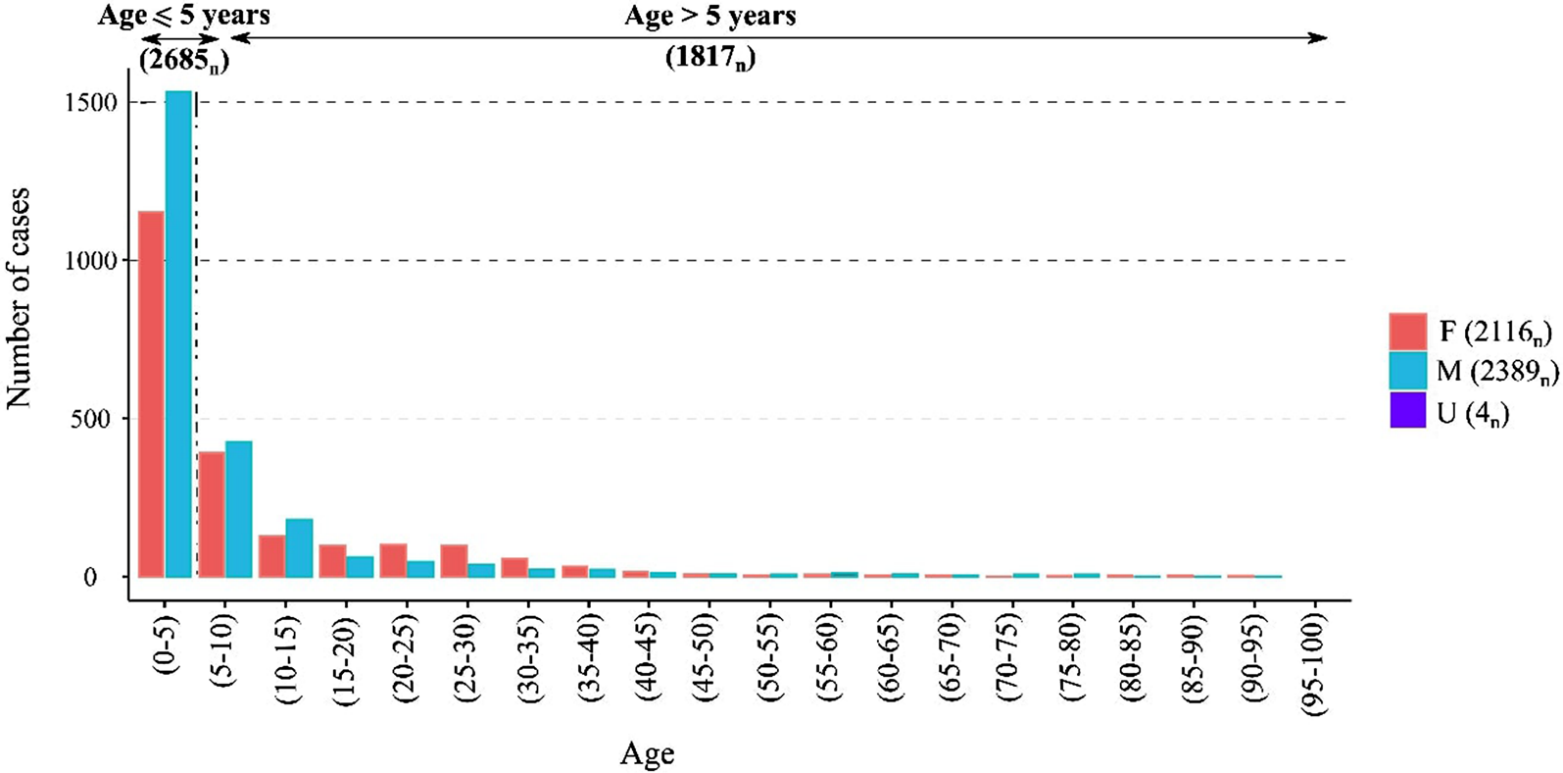
Age and gender distributions of cryptosporidiosis cases in the Republic of Ireland (2008–2017)

The dataset comprised 4,633 confirmed cases of cryptosporidiosis from 2008 to 2017, of which 4509 cases (97%) were successfully geo-linked to a distinct spatial unit (SA/ED centroid). Overall, 1964 Electoral Divisions (58% of 3,409), 3413 Small Areas (18.3% of 18,488) and all (26/26) Irish counties were associated with at least one confirmed case. Most cases were associated with children ≤ 5 years (n = 2672, 59.3%) (Fig. 2), with a slightly higher incidence rate reported among males (53%) (Table 1).

**Table 1:** Pearson χ^2^ test results for cryptosporidiosis cases in the Republic of Ireland, delineated by case type (sporadic, outbreak-related, travel), and age, gender and CSO classification

	Sporadic				Outbreak-related				Travel-related			
	Total cases	Odds Ratio	χ^2^	Sig	Total cases	Odds Ratio	χ^2^	Sig	Total cases	Odds Ratio	χ^2^	Sig
Gender												
F	1735 (45.6)	0.72	15.47	< 0.001	224 (54.8)	1.41	10.64	0.001	157 (52.5)	1.27	3.71	0.054
M	2066 (54.4)	1.38			185 (45.2)	0.71			142 (47.5)	0.78		
Case age												
≤ 5 Years	2325 (61.2)	1.51	25.5	< 0.001	166 (40.6)	0.99	0.007	0.932	181 (60.5)	0.41	53.08	< 0.001
> 5 Years	1476 (38.8)	0.66			243 (59.4)	1.01			118 (39.5)	2.39		
Classification												
Rural	2502 (65.8)	2.36	110.5	< 0.001	217 (53)	0.65	16.83	< 0.001	101 (33.8)	0.28		
Urban	1299 (34.2)	0.42			192 (47)	1.54			198 (66.2)	3.57		

**Fig. 3:**
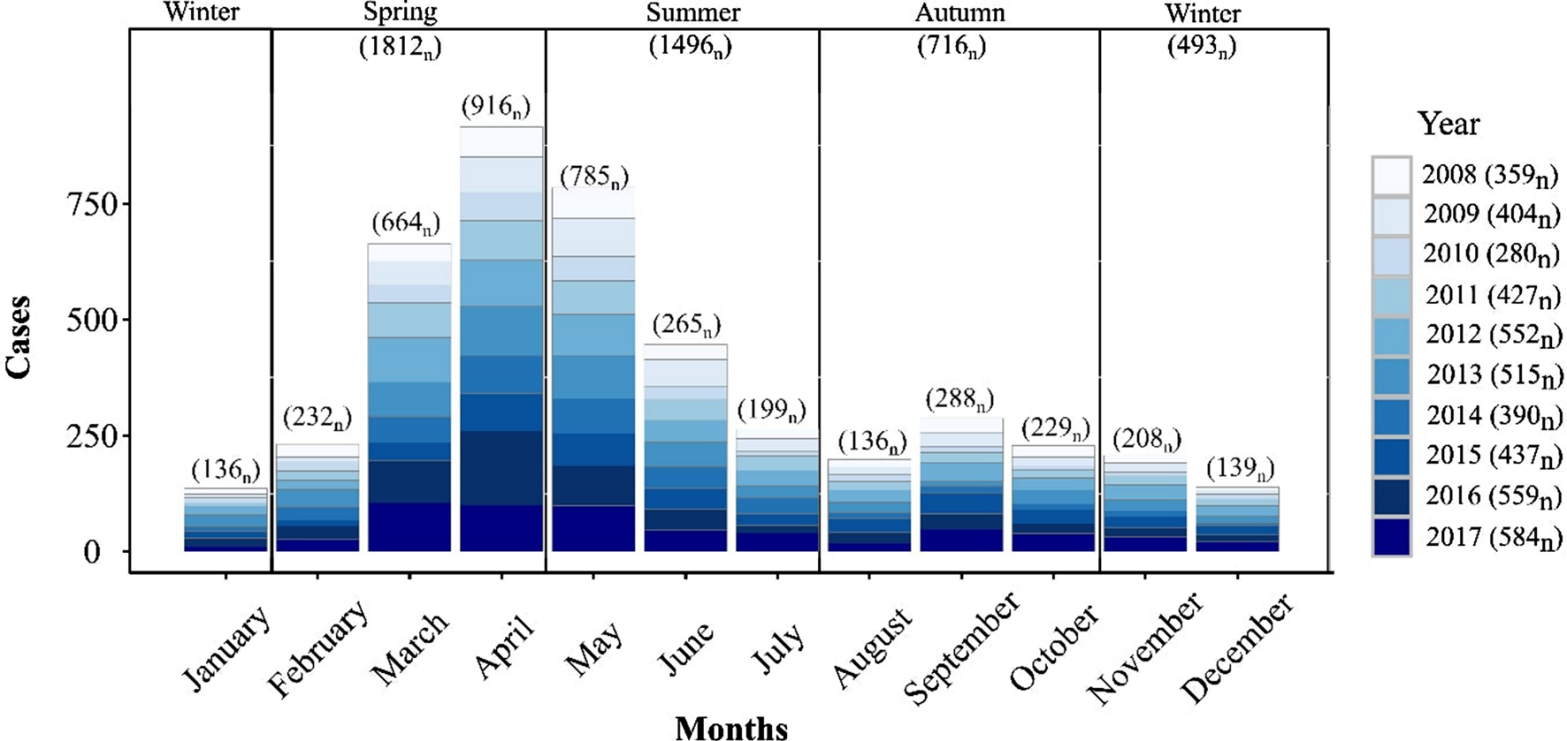
Temporal distribution of cryptosporidiosis cases in Ireland (2008–2017)

As shown (Table 1), sporadic cases were statistically more likely to be male (OR 1.4, 95% CI 1.2, 1.6), ≤ 5 years of age (OR 1.5, 95% CI 1.3, 1.8), and associated with a categorically rural area (OR 2.4, 95% CI 2, 28). Conversely, outbreak-related cases were associated with females (OR 1.4, 95% CI 1.2, 1.7) and urban areas (OR 1.5, 95% CI 1.3, 1.9). Travel-related cases were more likely to be female (OR 1.3, 95% CI 1, 1.6), > 5 years of age (OR 2.4, 95% CI 1.5, 3.1), and resident in an urban conurbation (OR 3.6, 95% CI 2.8, 4.6). Temporal cumulative incidence rates (Fig. 3) indicate a marked annual peak in late spring (n = 1812), with a maximum incidence rate occuring during April (n = 916). Lowest incidence rates were recorded during winter months (n = 493) with the lowest incidence rate being recorded in January (n = 136). Case numbers peaked in 2017 (n = 584).

### Seasonal decomposition

**Fig. 4:**
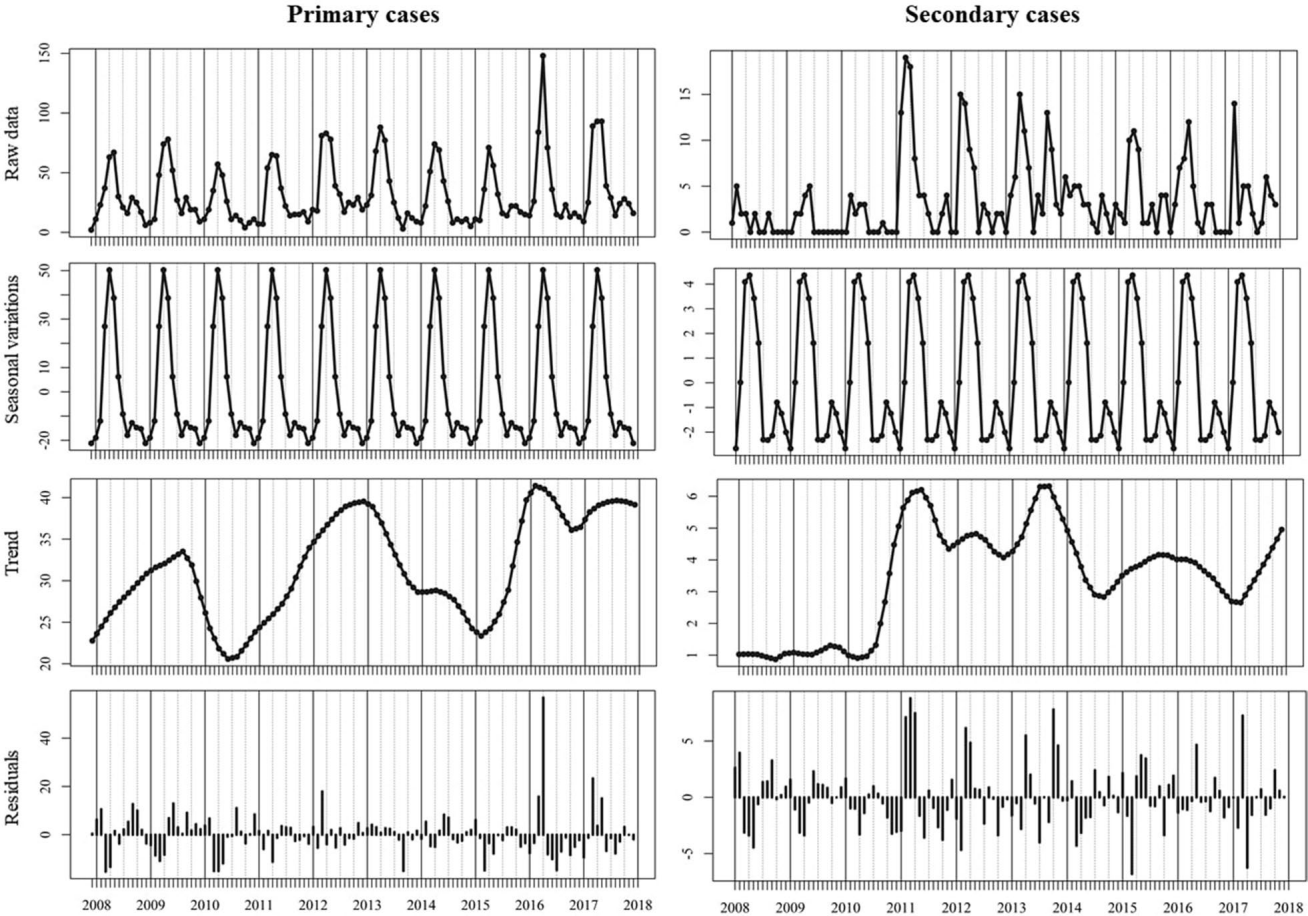
Seasonal decomposition of cryptosporidiosis in the Republic of Ireland (2008–2017), delineated by sporadic (left) and outbreak-related cases (right)

Seasonal decomposition of sporadic infection over the ten-year study period indicates a clear seasonal peak in mid-spring (April) annually (Fig. 4). Residual trends show a generally consistent annual and long-term trend with a notable peak of infection in April 2016 (Residual: + 56). Outbreak cases exhibit a similar seasonal trend to that of sporadic cases with annual peaks occurring in April, followed by a secondary peak in September. The overall long-term trend in outbreak cases displayed a marked increase during 2011, continuing until 2014. Residuals calculated for outbreak cases point to more variation in 10-year trends with peaks observed during the late winter/early spring months (January to March) of 2011, 2012 and 2017 while late spring/early summer peaks (April to June) were observed in 2013. A peak in outbreak-associated cases was also observed during the winter months (October to November) of 2013.

**Fig. 5:**
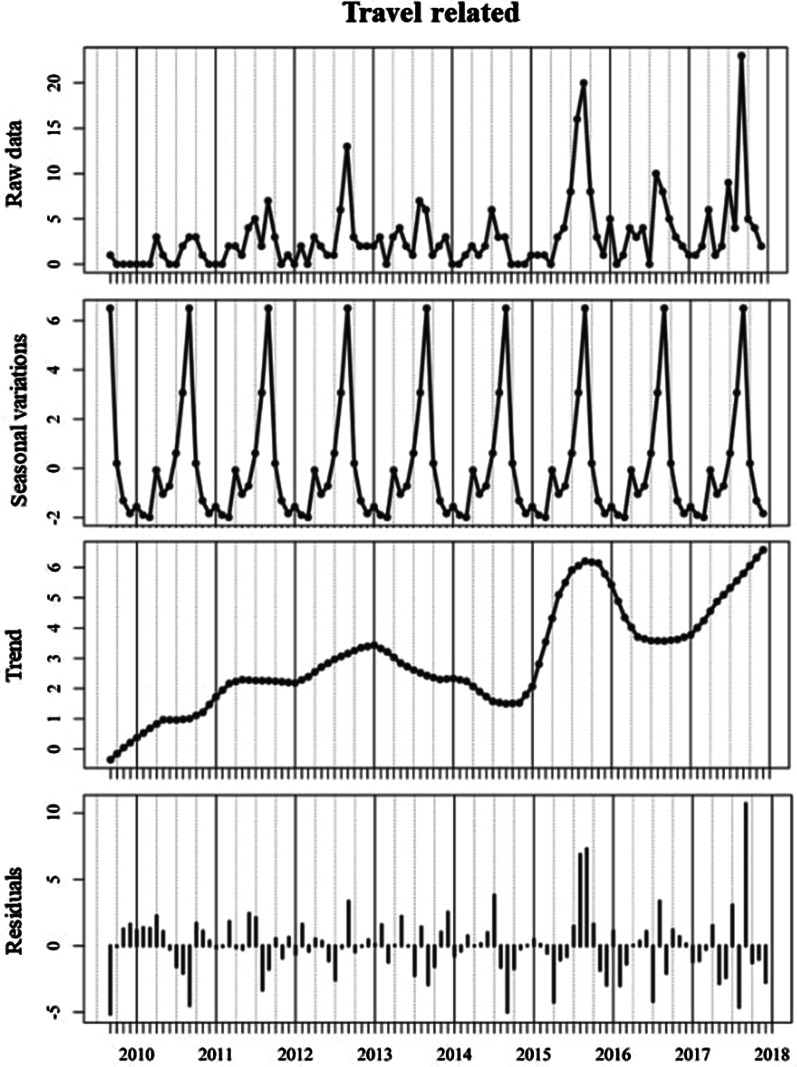
Seasonal decomposition of travel-related cryptosporidiosis in the Republic of Ireland (2008–2017)

**Fig. 6:**
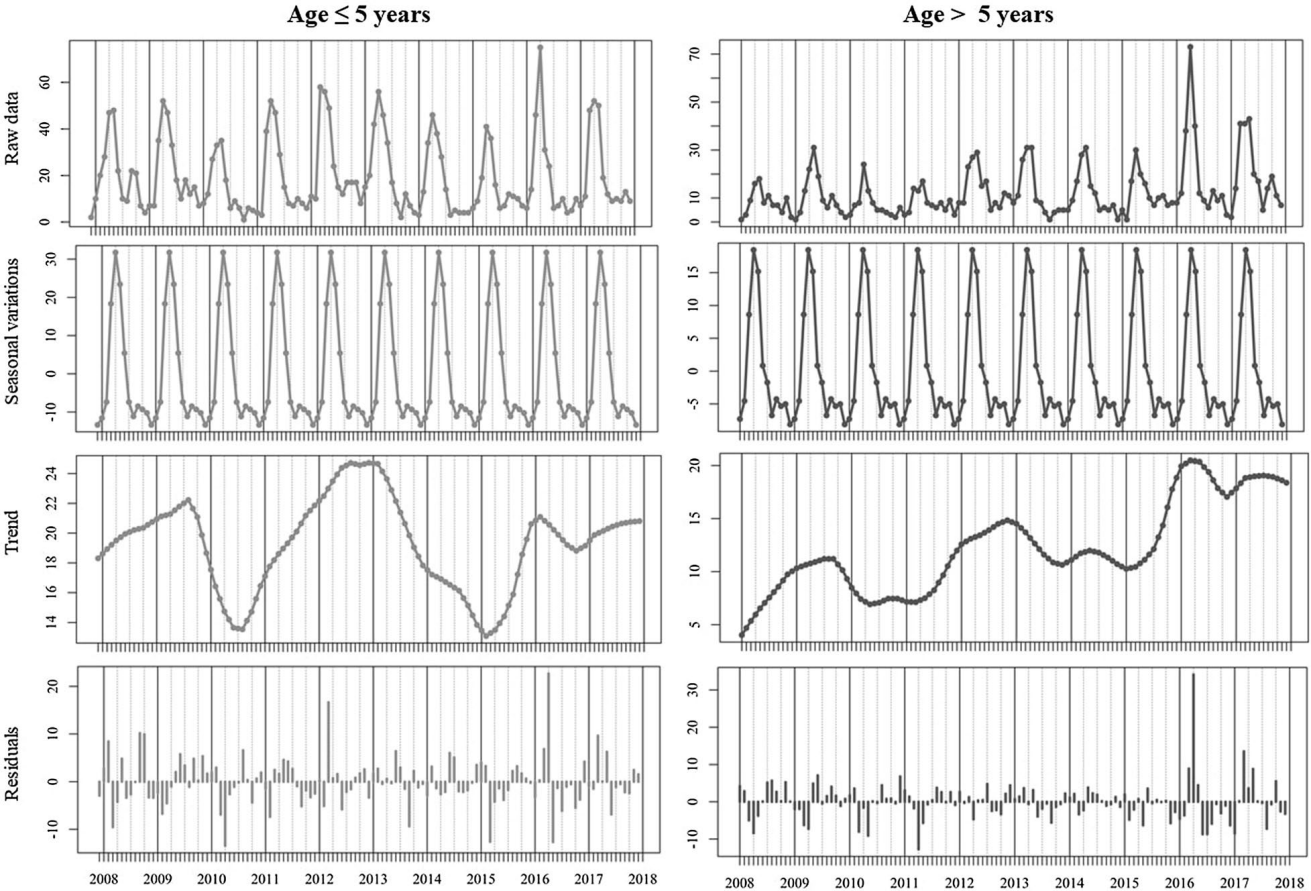
Seasonal decomposition of cryptosporidiosis in the Republic of Ireland (2008–2017), delineated by epidemiologically relevant age sub-categories

There was an increasing trend in the number/rate of travel-associated cases with an annual peak occurring in August/September (Fig. 5). The long-term trends varied significantly between delineated age categories with considerably more variation noted among children ≤ 5 years of age (Fig. 6), albeit annual peaks were observed among both age cohorts during April of each year. Residuals again point to a large transmission peak (Residuals: + 22, + 34) within both sporadic and outbreak cohorts during April 2016.

**Fig. 7:**
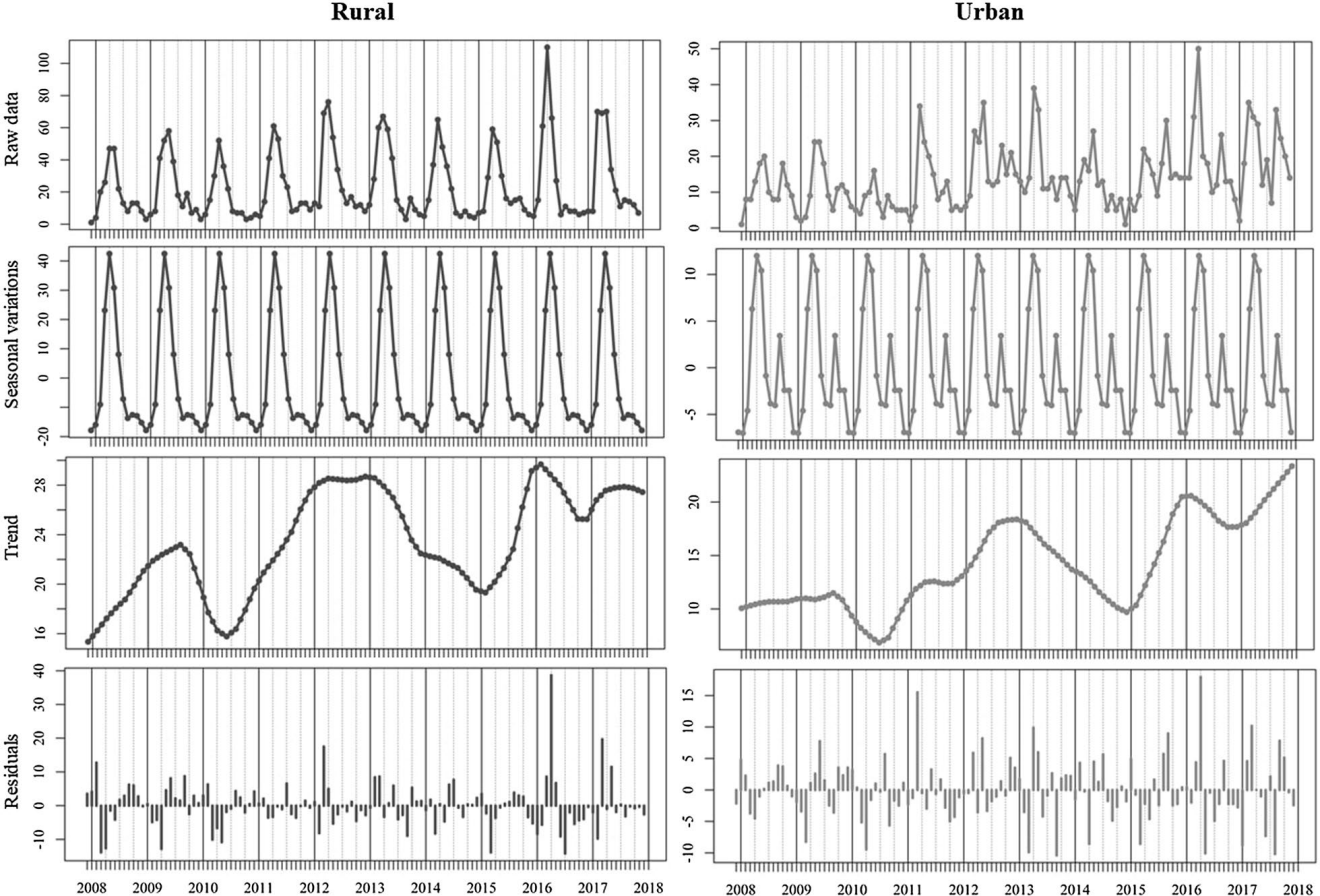
Seasonal decomposition of cryptosporidiosis in the Republic of Ireland (2008–2017), delineated by CSO urban/rural classification

Annual decomposed patterns of infection peaked in April of each year followed by a significantly smaller peak during September in both urban and rural areas (Fig. 7). Calculated residuals point to an infection peak in April 2016 in both urban (+ 18) and rural (+ 38) areas, consistent with trends observed among sporadic and age-delineated infection peaks.

### Spatial autocorrelation

**Fig. 8:**
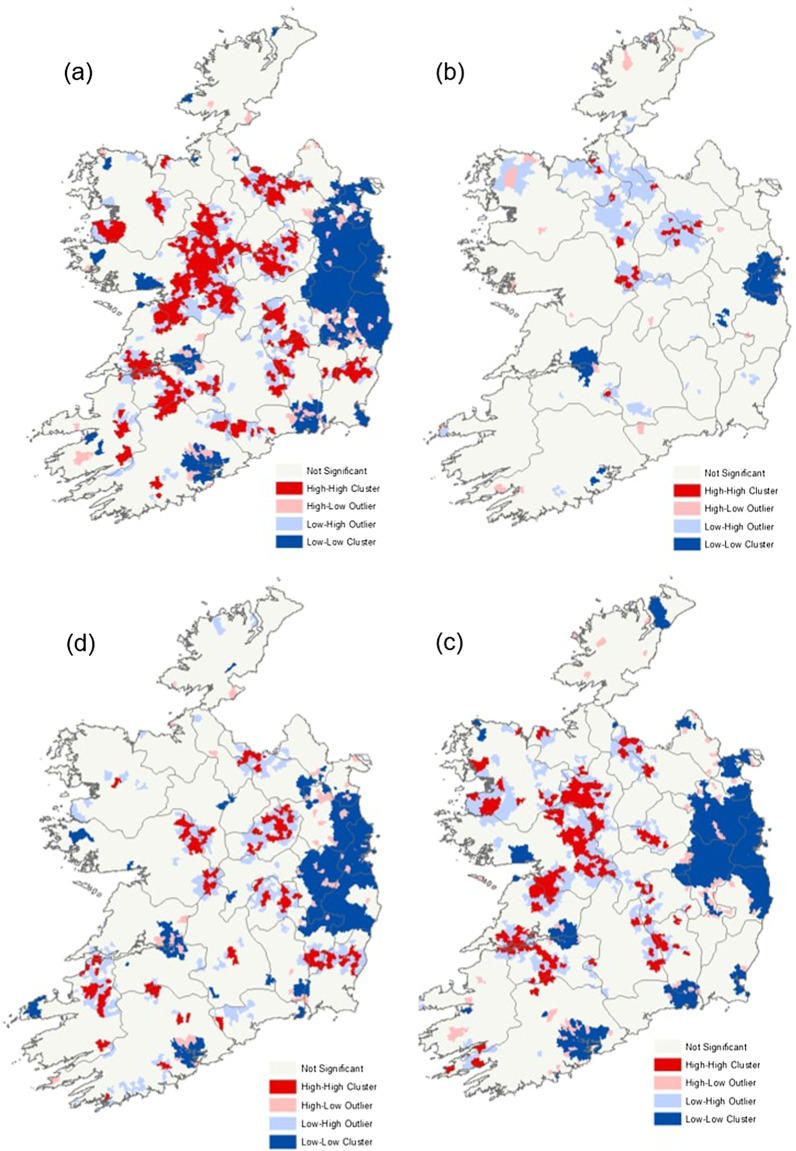
**a** Sporadic cryptosporidiosis case clusters and outliers determined by Anselin Local Moran’s I clusters **b** Outbreak-related cryptosporidiosis case clusters and outliers determined by Anselin Local Moran’s I clusters **c** Sporadic cryptosporidiosis case clusters and outliers among children aged 5 years and younger determined by Anselin Local Moran’s I clusters **d** Sporadic cryptosporidiosis case clusters and outliers among the cohort of people age 6 years and older determined by Anselin Local Moran’s I clusters

A significant H–H cluster of sporadic cases was observed in the midland (M) region, with a large L–L cluster identified along the eastern seaboard (E), surrounding the greater Dublin urban area and commuter belt (Fig. 8a). L–L clusters were also observed in the S and SE regions, spatially proximal to the urban conurbations of Cork, Waterford, and Limerick cities. Smaller H–H clusters of infection were observed in the S, SE and W regions of the country, consistent with an overarching urban/rural pattern. Notable L–L outbreak-related case clusters were observed in the east of the country surrounding Dublin city and in the south surrounding Limerick city (Fig. 8b). Few H–H clusters were associated with outbreak-related cases, however H–H cases identified in the midland region (M) were surrounded by L–H clusters, thus indicating potential neighbouring outliers. H–H and L–L clusters of infection in children ≤ 5 followed a broadly similar spatial pattern to that observed within the sporadic case cohort, due to the large proportion of cases from this cohort comprising the total dataset (Fig. 8c). A large H–H cluster was observed in the M region, with smaller H–H clusters again identified in S, SE and W regions. L–L clusters of infection were also consistent with sporadic case clusters and typically identified around urban areas in the S and SE of the country. The spatial predominance of infection cold spots (L–L) among people age > 5 (Fig. 8d) followed a similar pattern of infection cold spots among sporadic cases and paediatric (≤ 5 years) cases (Fig. 8c). However, the spatial predominance of infection hot spots among this cohort was markedly different to sporadic and ≤ 5-year hot spots, with smaller and more spatially dispersed hot spots identified, primarily in the midlands (region M) and SW regions.

### Hot-spot analysis (Getis-Ord GI*)

**Fig. 9:**
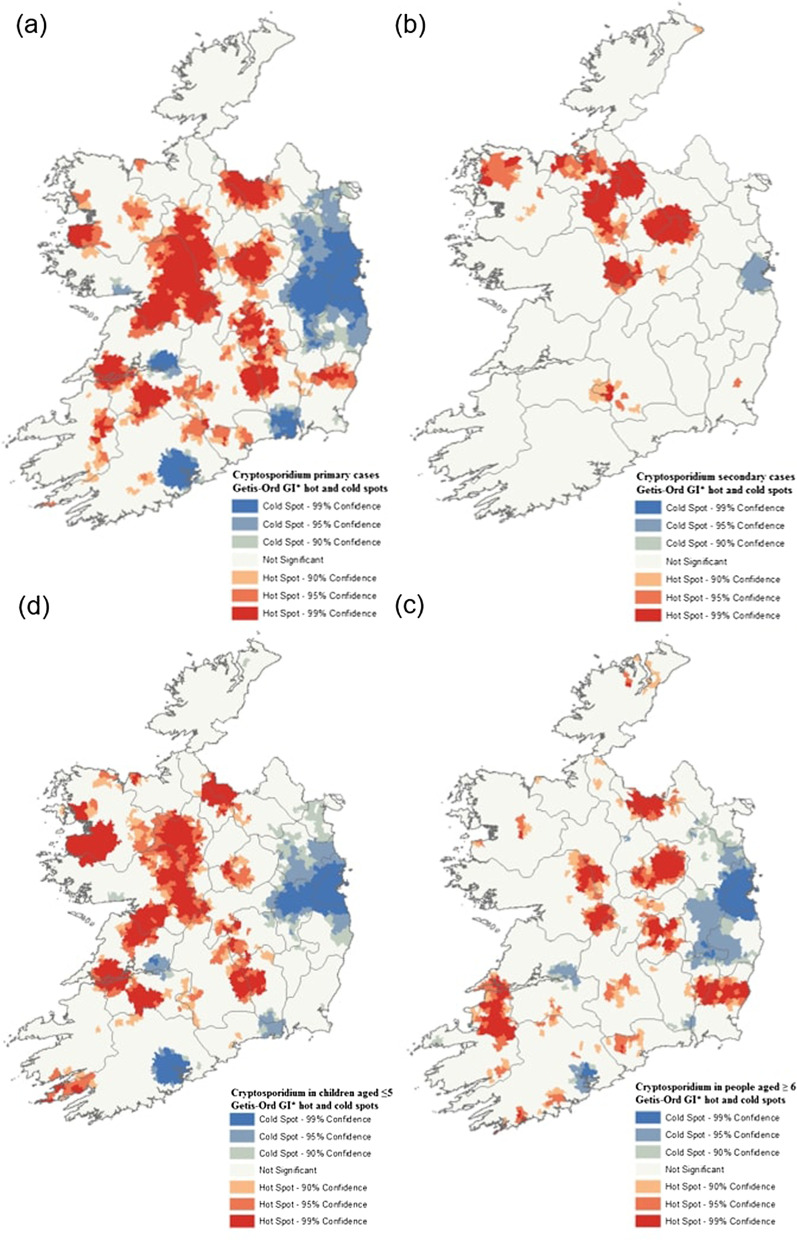
**a** Sporadic cryptosporidiosis case hot and cold spots determined by Getis-Ord Gi* hot-spot analysis—**b** Outbreak-related cryptosporidiosis hot and cold spots determined by Getis-Ord Gi* hot-spot analysis—**c** Sporadic cryptosporidiosis case hot and cold spots among children aged 5 years and younger determined by Getis-Ord Gi* hot-spot analysis—**d** Sporadic cryptosporidiosis case hot and cold spots among the cohort of people age 6 years and older determined by Getis-Ord Gi* hot-spot analysis

Getis-Ord GI* analyses identified notable hot spots among sporadic cases in the midlands (M), east and north-east of Galway city, with smaller hot spots also evident in the midlands, south and south-east (M, S and SE) (Fig. 9a). Again, a spatially extensive cold spot was identified in the east of the country (E), encompassing the greater Dublin metropolitan urban area, and in the south and south-east (S and SE) around Waterford, Limerick and Cork cities.

The spatial predominance of hot and cold spots among children ≤ 5 years again followed a similar pattern to clustering of infection among all sporadic cases (Fig. 9c). Large hot spots were observed in the midlands and south (M and S), with a previously identified sporadic infection hot spot in the west (W) demonstrating a significantly more pronounced occurrence among the paediatric subpopulation (NE of Galway city). A large cold spot among children ≤ 5 was also observed in the in the greater Dublin area (E), albeit significantly reduced when compared with that observed among all sporadic infections. The spatial distribution of hot and cold spots of infection among people aged > 5 varied (Fig. 9d), with the spatial distribution of hot and cold spots of sporadic infection and infection in children ≤ 5. One hot spot was identified in the SW region, which was not observed using other statistical methods or among other subcategories of infection.

### Space–time scanning

**Fig. 10:**
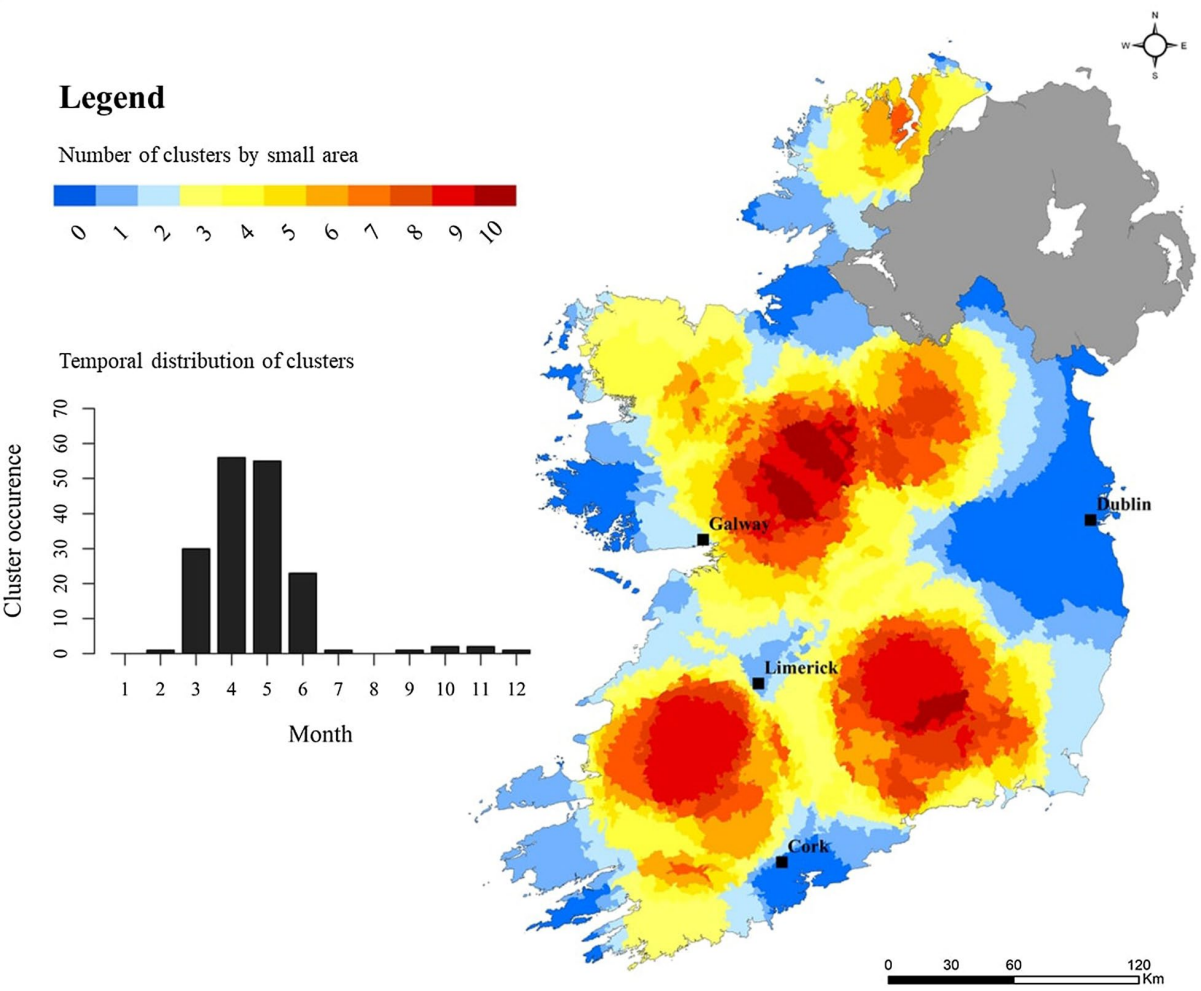
Space–time “cluster recurrence” index (0–10) for sporadic cryptosporidiosis cases in the Republic of Ireland, 2008–2017

Space–Time clustering recurrence and cluster temporality for sporadic cryptosporidiosis cases are presented in Fig. 10, with results of year-on-year space–time scanning presented in Additional file 1: Appendix 1. Annual space-time clusters of Cryptosporidiosis in Ireland from 2008 to 2017; Appendix 2. Space-time clusters of Cryptosporidiosis in Ireland during 2008. As shown (Fig. 10), three primary hot spots were identified: south-west and east of Limerick city (SW, S, SE), and north-east of Galway city (M). Cold spots are persistent along much of the eastern seaboard, and particularly around the larger urban conurbations of greater Dublin and Cork city, in addition to significant areas of the western coastline. The temporal window for space–time clusters mirrors the general seasonal distribution of cryptosporidiosis infection (Sect. 3.2), with peak cluster identification occurring from March to June and peaking in April.

**Fig. 11:**
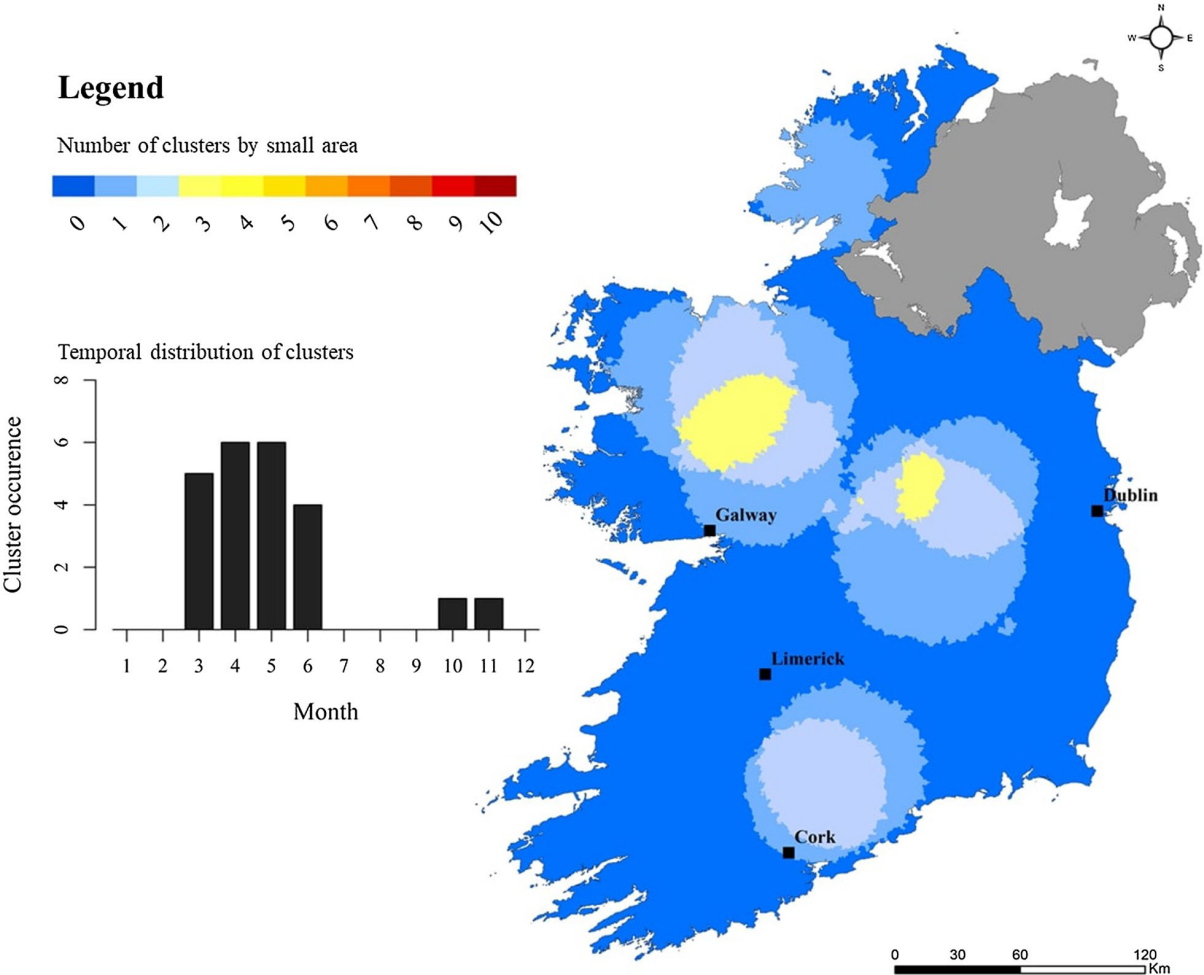
Space–time “cluster recurrence” index (0–10) for outbreak-related cryptosporidiosis cases in the Republic of Ireland, 2008–2017

**Fig. 12:**
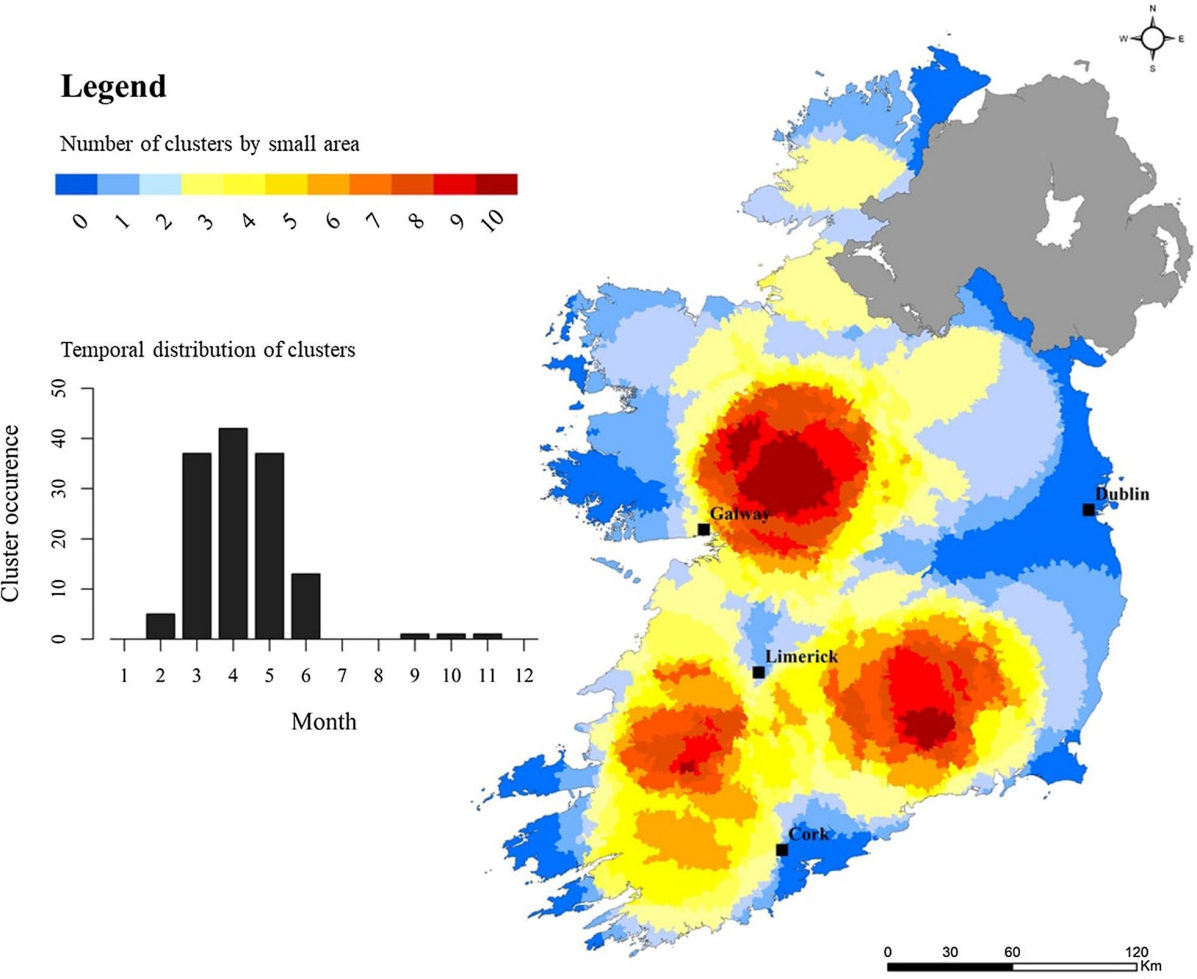
Space–time “cluster recurrence” index (0–10) for sporadic cryptosporidiosis cases in the Republic of Ireland, 2008–2017 (Delineated by epidemiologically relevant age category—Population ≤ 5 years)

**Fig. 13:**
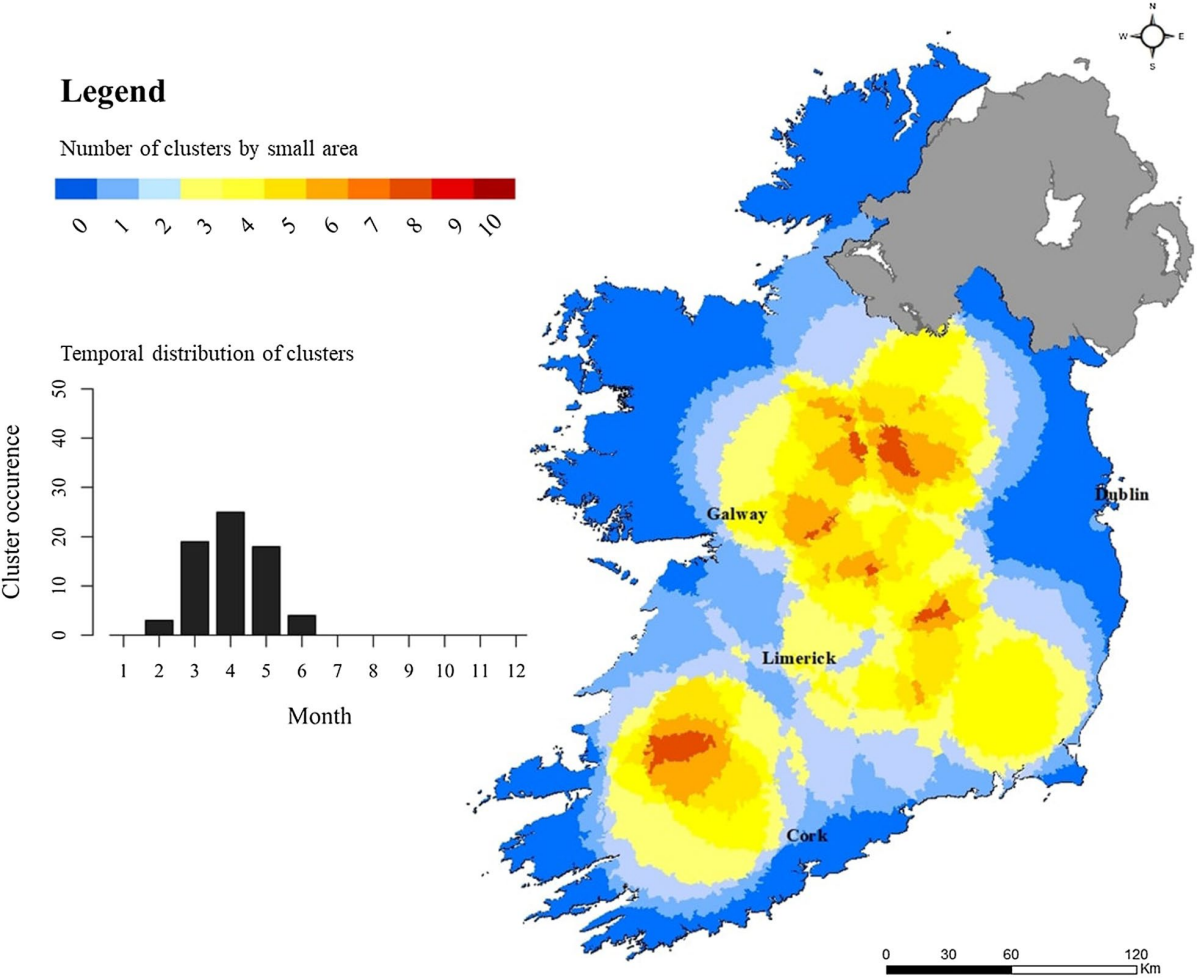
Space–time “cluster recurrence” index (0–10) for sporadic cryptosporidiosis cases in the Republic of Ireland, 2008–2017 (Delineated by epidemiologically relevant age category—Population > 5 years)

Significantly lower levels of space–time clustering were found among outbreak-related cases (Fig. 11), with largest hot spots located in western and midland regions (M, W), and a maximum cluster recurrence of 30% (i.e., geographic area included in 3 identified clusters over 10 annual iterations). Two additional space–time clusters were identified to the north-east of Cork city (S) and County Donegal (N). Most (8/9) outbreak-related clusters were observed from March to June, with one cluster occurring during October/November (2013). Cluster index mapping for ≤ 5 year sub-population mirrored that of sporadic cases, with three primary hot spots identified; again, a large area located north-east of Galway city (M), and two “secondary” (i.e., lower cluster recurrence indices) areas located south-west and south-east of Limerick city (SW,S) (Fig. 12). Results for the sub-population > 5 years point to a lower level of clustering, with hot spots located south-west of Limerick city (SW), the Midlands (M) and south-east (SE) (Fig. 13).

## Discussion

### Occurrence of sporadic and outbreak-associated cryptosporidiosis

Cryptosporidiosis exhibits a relatively wide geographical distribution in Ireland with 58% and 18.3% of Electoral Divisions and Small Areas associated with at least one confirmed case during the study period, respectively. Crude Incidence Rates (CIRs) of infection indicate a moderately increasing trend, ranging from 9.8/100,000 in 2008 to 12.4/100,000 in 2017 [10]. Most (59.3%) sporadic cases were associated with children ≤ 5 years, which concurs with several previous studies [3, 7]. Within the ≤ 5 years cohort, cases were more frequently associated with male children (OR 1.3873), potentially reflecting the tendency of male children to mount weaker immune responses [21], an enhanced susceptibility to environmental exposures via gender-related outdoor activities [22], or a gender-related bias in healthcare-seeking behaviours [23]. Conversely, female children were statistically associated with outbreak-related cryptosporidiosis, potentially reflecting higher levels of direct contact (and subsequent transmission) between parents/family members and female children [24]. A recent small-scale investigation of the regional epidemiology of cryptosporidiosis in County Cork, Ireland, demonstrated moderately increased infection rates among 20–34-year olds, suggesting likely anthroponotic transmission via caregiver contact with infected children [25]. Geographically, most sporadic cases (65.8%) occurred in categorically rural areas (χ^2^ = 110.493, p < 0.001; Table 1), where approximately 37.3% of the Irish populace reside [26]. A previous Scottish study by Pollock et al. similarly found *C. parvum* infection was associated with areas characterised by lower human population density and a higher ratio of farms to humans, both indicators of rurality [27]. While the current study represents the first nationwide study of the spatiotemporal epidemiology of cryptosporidiosis in Ireland, this finding was expected, and likely attributable to increased exposure to sources of *Cryptosporidium* spp. oocysts in rural areas, including farmyard animals [28], direct exposure to contaminated surface waters [29] and the use of groundwater as a drinking water source [6]. Conversely, urban areas exhibited a significantly higher secondary (OR 1.5383) and travel-related (OR 3.5742) case occurrence, likely indicative of *C. hominis* infections as opposed to the agriculturally (rural) associated *C. parvum*, however, as *Cryptosporidium* spp*.* is not identified within the Irish disease reporting system, this is somewhat speculative.

### Seasonal decomposition

Seasonal decomposition points to an overall increasing temporal trend over the ten-year study period (Fig. 4), consistent with previously reported trends in the west of Ireland during 2004–2007 [30]. Specifically, the annual peak found during April is consistent with previously reported regional peaks (March/April) [30], in addition to those reported in Scotland (April/May) [27], likely associated with agricultural cycles in temperate regions i.e. lambing/calving and manure spreading. While not reported in the current study, seasonal patterns may vary among differing *Cryptosporidium* species; for example, *C*. *hominis* is more prevalent during autumn in the UK and New Zealand (increased travel and school/childcare attendance), whereas *C.* *parvum* is more typically encountered during spring in Canada, Ireland and The Netherlands [9]. The secondary peak observed among outbreak-related cases during September (Fig. 4) is consistent with the bimodal peaks observed in *C. hominis* in Scotland in August and October [27], and may reflect the increase in national/international travel and children returning to childcare/school after summer break.

Seasonal decomposition also identified several notable deviations (i.e., residual peaks) from the overarching temporal trend which merit closer investigation, particularly regarding dynamic drivers of exposure/transmission such as extreme weather events [28, 31]. A marked positive residual was identified during April 2016 (Fig. 4), initiating further exploration with respect to dynamic meteorological events, particularly in light of severe flooding experienced across Ireland and the UK [32]. Winter 2015/2016 was characterised by a succession (n = 6) of Atlantic storms across Ireland, resulting in exceptional and widespread flooding with all synoptic weather stations reporting rainfall volumes significantly above their Long-Term Average (LTA) [32]. Recent work by Boudou et al. have shown that excess cases of cryptosporidiosis were widespread during and after the flood period, with areas characterised by the presence of a surface water body exhibiting significantly higher incidence rates (OR 1.363; p < 0.001) [32]. Time-series modelling of the event presented a clear association between rainfall, surface water discharge, groundwater levels and infection incidence, with lagged associations from 16 to 20 weeks particularly strong, thus indicating a link between infection peaks (April 2016) and the flood event which began approximately 18 weeks earlier [32]. Thus, it was concluded that increases in storm water, soil saturation and surface runoff increased pathogen mobility for a significant period, thus exacerbating transmission of cryptosporidiosis both directly (i.e., contaminated ‘raw’ water and food) and indirectly (i.e., long-term soil saturation) [32]. Similarly, a cryptosporidiosis outbreak which occurred during August 2013 in Halle, Germany, began six weeks after the river Saale inundated the floodplain and parts of the city centre [3], thus emphasising the (lagged) impact of local meteorological conditions on the incidence of infection.

### Spatial autocorrelation and Hot-Spot analysis

Incorporating a spatial dimension into investigations of infectious disease epidemiology is of primary importance considering the spatial variation of environmental exposure such as land use, local climate, and socioeconomic status, particularly in Ireland which has previously been described as “the perfect storm” with regard to potential gastroenteric infection risk factors [33]. Results of Anselin Local Moran’s *I* statistics and the Getis Ord GI* statistic provided relatively similar spatial patterns. High incidence (H-Hs) clusters were identified in the Irish Midlands (M), a predominantly rural area with a high level of dependence on pastoral agriculture and “private infrastructure” (e.g., one-off housing with on-site wastewater treatment and domestic water supplies). Several previous studies have documented strong associations between cryptosporidiosis and cattle density [27, 34]. Similarly, a study from central Wisconsin previously found the incidence of endemic diarrhoeal infections significantly higher in areas characterised by elevated septic tank (OR 1.22) and private water supply (OR 6.18) densities among a population-based cohort [35]. Conversely, low incidence (L-L) clusters were primarily located in the vicinity of Ireland’s capital (Dublin) and other relatively large cities (Waterford, Cork, Limerick, Galway), thus likely highlighting the protective effect of urban living within the Irish context, where reduced environmental exposure to pathogen sources coupled with reduced pathogen transport (i.e., treated drinking water supply) may reduce the risk of exposure and subsequent infection [36]. Conversely, recent studies have shown rates of cryptosporidiosis are typically higher in urban areas characterised by elevated human population densities, for example Cohen et al. previously reported that higher population density and above average household sizes were associated with increased odds of reported cases of cryptosporidiosis in Massachusetts [37]. Likewise, Greenwood & Reid have found that most cryptosporidiosis clusters identified across Queensland, Australia from 2001 to 2015 centred on major and regional cities [38].

Both geostatistical techniques suggest a disparity exists with respect to outbreak-related clustering over the 10-year study period, as they relate to clustering of sporadic cases (Figs. 8a, b, 9a, b), with outbreak-related clusters occurring in the north Midlands and “border area”, regions traditionally characterised by relatively low population densities. This merits further investigation within the context of population age structure, household size and domestic water source, along with close monitoring and surveillance by the relevant Departments of Public Health.

### Space–Time scanning and cluster recurrence

Space–time scan statistics detect temporally-specific clusters characterised by a significantly higher observed case number than expected (e.g., space–time randomness not present), based on calculated baseline incidence rates [20] with the approach employing a 3-dimensional (cylindrical) scanning window comprising both height (time) and space (geographic area) [19]. Over the past decade, space–time scan statistics have been recognised as a powerful tool for endemic disease surveillance and early outbreak detection [39], however to the authors knowledge, this represents the first time it has been applied to infectious disease incidence in Ireland. A total of 69 space–time clusters (≥ 10 confirmed cases) were identified over the 10-year study period, of which 55 (79.7%) were clusters of sporadic infection, ranging from a minimum of 4 (7.3%) during 2017 to a maximum of 7 (12.7%) during 2009. No statistical association was found between annual sporadic and outbreak-related cluster number during the study period, however development of the “cluster recurrence” index (e.g., Figs. 10, 11, 12, 13) permits identification of discernible spatial and temporal patterns defining the formation of clusters across the decade-long period. Three regions exhibited particularly recurrent space–time clusters of infection, with occurrences during ≥ 8 out of 10 years, namely south-west and east of Limerick city (SW, S, SE), and north-east of Galway city (M), with neither urban conurbation actually located within a high recurrence region. The spatiotemporal frequency of space–time clusters suggests the presence of persistent reservoirs in these areas thus maintaining community and/or transmission pathways [38]. The proximity of large urban centres to each high-recurrence region may potentially reflect relatively narrow transitional zones between urban fabric and populated rural regions i.e., rural commuter belts which remain un-serviced with respect to municipal wastewater treatment and/or drinking supplies. Additionally, all three regions are predominantly underlain by karstified carboniferous limestone aquifers [40] which have previously been associated with the presence of *Cryptosporidium* spp. in private and small public drinking water supplies [12, 41]. Conversely, the Greater Dublin area, characterised by a large urban commuter belt, spatially extensive consolidated bedrocks and high levels of municipal water and wastewater infrastructure, did not exhibit any space–time clusters over the study period. A significant majority of space–time clusters occurred over the 4-month period May–June, thus mirroring findings from the overall case cohort, and further highlighting the likely association between agricultural cycles and the incidence of infection in temperate regions including Ireland, Scotland and New Zealand [27, 42]. Additionally, Lal et al. have signalled a need to study the effect of spatial and temporal variations in ecological and social risk factors on the incidence of cryptosporidiosis with specific emphasis on the potential for socioeconomic disadvantage to amplify disease risk within populations, e.g., in areas of low educational attainment and lower income levels, which are often associated with rural living [28].

From a public-health surveillance perspective, identification of 55 space–time clusters of sporadic cryptosporidiosis infection over a 10-year period represents a concern, while underscoring the major challenges involved in decreasing the incidence of infection via enhanced surveillance and subsequent intervention. For example, during 2008, a spatially restricted space–time cluster which was identified in the northern Midlands (Cluster 2, Additional file 1: Appendix) was characterised by almost 18 times more cases of infection than would be expected (RR 17.95) over a three-month period (February–April), with several identified space–time clusters occurring over time periods as short as 4 weeks. As such, this level of clustering may suggest the need for new surveillance and/or analytical methods to elucidate hitherto unidentified sources and pathways of infection, and to identify space–time clusters while they exist i.e., real-time or prospective scanning [43].

It is important to note that a lack of species information, and particularly the inability to discern between *C. parvum* and *C*. *hominis*, the two most frequently encountered *Cryptosporidium* species in Ireland, represents a study limitation. As previously outlined, Pollock et al. found *C. parvum* infection to be associated with lower population density and higher ratio of farms to humans, indicators of rurality, while *C. hominis* was more likely to be found in the more urban area of southern Scotland [27]. Speciation would thus permit closer elucidation of sociodemographic influences on rural/urban distribution. Further investigation is required to elucidate potential sources and pathways of infection, with particular regard to livestock densities, climate, hydrogeology and socioeconomic status.

In conclusion, despite mandatory surveillance of cryptosporidiosis due to its communicable disease status in Ireland, it is widely regarded that cryptosporidiosis remains under-reported in Ireland and on a broader European level. The spatiotemporal epidemiology of cryptosporidiosis in Ireland reflects the diverse population and geography of the country, albeit with a markedly higher rate of occurrence in rural areas, likely due to the ubiquity of *Cryptosporidium* spp. sources (e.g., cattle) and pathways (e.g., karstic limestone bedrocks). The elevated burden among children ≤ 5-years is likely related to both immunological status and specific routes of exposure and warrants further study. The presented study represents a significant advance in efforts to investigate the spatiotemporal epidemiology of cryptosporidiosis with a view to further elucidating pathways of infection to guide public-health interventions through an improved understanding of its spatio-temporal occurrence, clustering mechanisms, levels of recurrence, and associated drivers, pathways, and receptors.

## Supplementary Information

**Additional file 1. Appendix 1**. Annual space-time clusters of Cryptosporidiosis in Ireland from 2008 to 2017;** Appendix 2**. Space-time clusters of Cryptosporidiosis in Ireland during 2008.

## Data Availability

Due to the sensitive nature of the study data, datasets are not publicly available. For further information related to data acquisition, please contact the corresponding author, Dr Paul Hynds (email: Paul.Hynds@tudublin. i.e., phone: 0,838,256,888).
