# Supplementary material for: Spatiotemporal epidemiology of cryptosporidiosis in the Republic of Ireland, 2008–2017: development of a space–time “cluster recurrence” index
Source: BMC Infect Dis. 2021 Aug 28;21:880. doi: 10.1186/s12879-021-06598-3 (PMC8401175; doi:10.1186/s12879-021-06598-3)
Supplement: Supplementary file 1 — Additional file 1. Appendix 1. Annual space-time clusters of Cryptosporidiosis in Ireland from 2008 to 2017; Appendix 2. Space-time clusters of Cryptosporidiosis in Ireland during 2008. [file 12879_2021_6598_MOESM1_ESM.docx]

**Supplementary materials**

# Title : Spatiotemporal epidemiology of cryptosporidiosis in the Republic of Ireland, 2008-2017

**European Journal of Epidemiology**

Boudou M.^1^, Cleary E.,^1^ ÓhAiseadha C.,^2^ Garvey P.,^3^ McKeown P.,^3^ O’Dwyer J*.,^4,5^ Hynds P.*^1,5^

**^1^** Environmental Sustainability & Health Institute (ESHI), Greenway Hub, Grangegorman, Dublin 7, Republic of Ireland

**^2^** Department of Public Health, Health Service Executive (HSE), Dr. Steevens’ Hospital, Dublin 8, Republic of Ireland

^3^ Health Protection Surveillance Centre, 25 Middle Gardiner Street, Dublin 1, Republic of Ireland

^4^ School of Biological, Earth and Environmental Sciences, Environmental Research Institute (ERI),

University College Cork, Cork, Republic of Ireland

^5^ Irish Centre for Research in Applied Geosciences (iCRAG), University College Dublin, Dublin 4, Republic of Ireland

* Corresponding Authors: [hyndsp@tcd.ie](mailto:hyndsp@tcd.ie), [jean.odwyer@ucc.ie](mailto:jean.odwyer@ucc.ie)


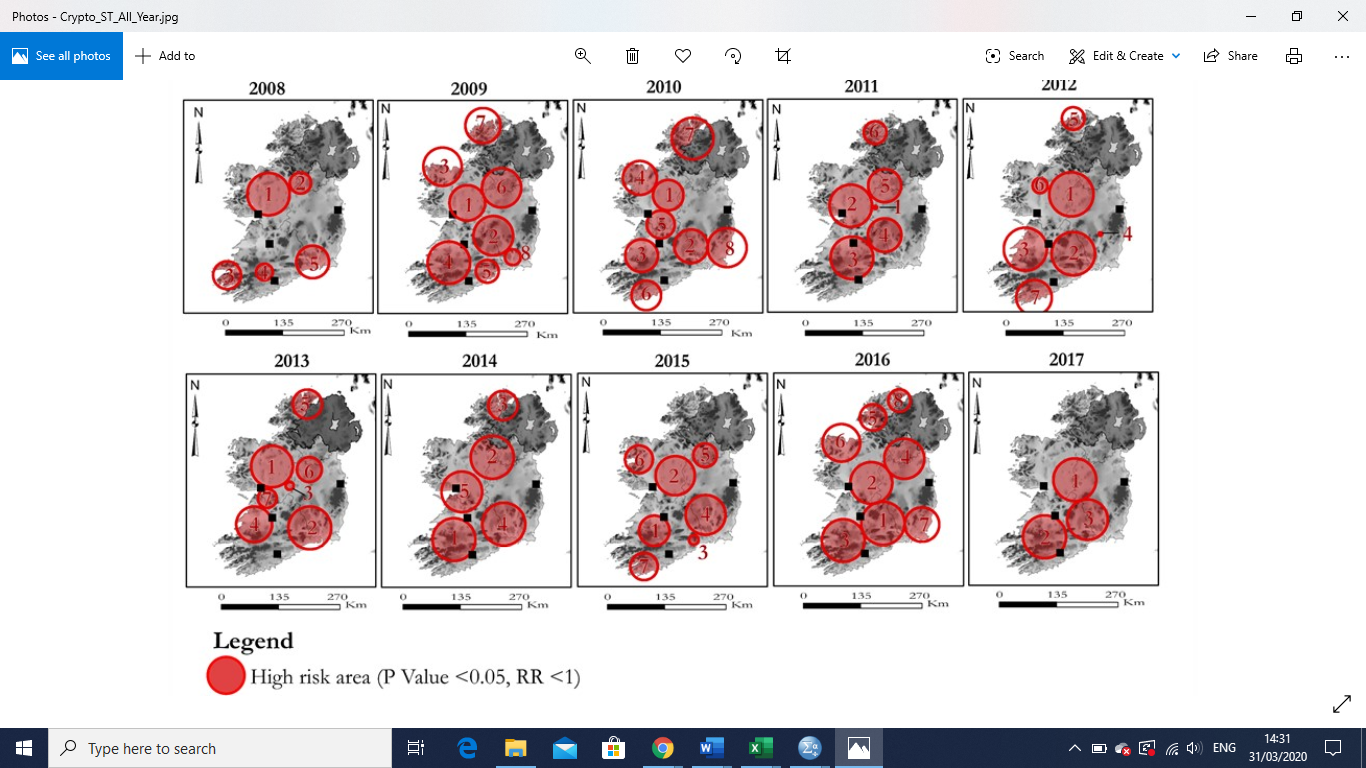


Appendix 1. Annual space-time clusters of Cryptosporidiosis in Ireland from 2008 to 2017


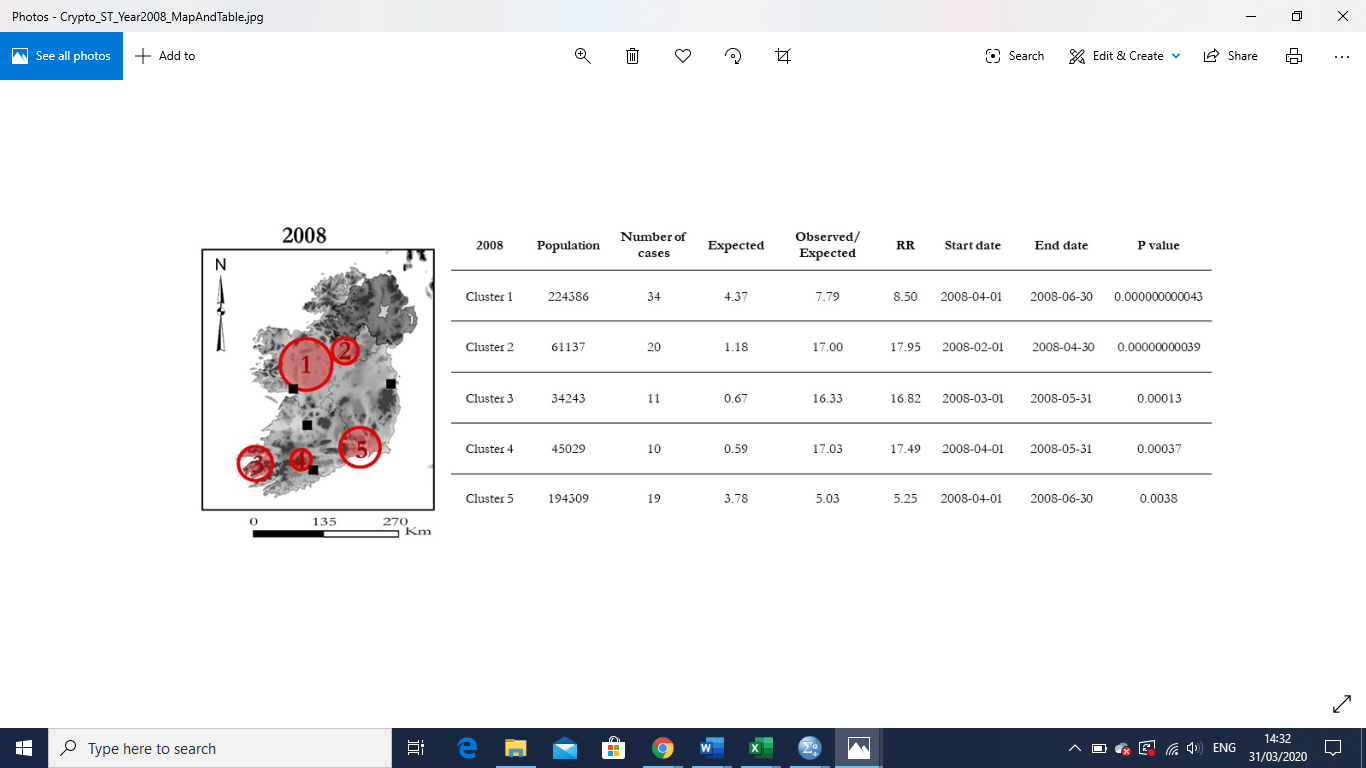


Appendix 2. Space-time clusters of Cryptosporidiosis in Ireland during 2008
